# Determination of the optimal location for constructing solar photovoltaic farms based on multi-criteria decision system and Dempster–Shafer theory

**DOI:** 10.1038/s41598-020-65165-z

**Published:** 2020-05-18

**Authors:** Marzieh Mokarram, Mohammad J. Mokarram, Mohammad R. Khosravi, Ali Saber, Akbar Rahideh

**Affiliations:** 1 Department of Range and Watershed Management, College of Agriculture and Natural Resources of Darab, Shiraz University, Shiraz, Iran; 2 Department of Electrical and Electronic Engineering, Shiraz University of Technology, Shiraz, 71555-313 Iran; 3 Telecommunications Group, Department of Electrical and Electronic Engineering, Shiraz University of Technology, Shiraz, 71555-313 Iran; 4 Department of Civil and Environmental Engineering and Construction, University of Nevada Las Vegas, 4505 S. Maryland Pkwy., Las Vegas, NV 89154 USA; 5 Department of Computer Engineering, Persian Gulf University, Bushehr, Iran

**Keywords:** Electrical and electronic engineering, Energy grids and networks

## Abstract

Considering environmental concerns regarding air pollution which is induced by burning fossil fuels to generate electrical power, utilizing solar energy as a green and sustainable energy source is of great interest. This study proposes a novel framework to determine the optimal location for constructing solar photovoltaic (PV) farms. To locate the suitable areas for PV farms, firstly, a fuzzy-based method is utilized to homogenize the input parameters, thereafter, the analytical hierarchy process (AHP) and Dempster-Shafer (DS) methods are independently used. In the AHP method, the proper weight for each input parameter is generated utilizing a pairwise comparison matrix. However, the DS method identifies output in different confident levels. Finally, southeast of Fars province in Iran as a region with high sunny hours in the year is selected, and the applicability of proposed methods is examined. The results show that 32% of the case study is located at high and good suitability classes in the fuzzy_AHP method. However, it is 18.56%, 16.70%, 16.32% according to 95%, 99% and 99.5% confident levels in the fuzzy_DS method, respectively. Comparisons of the fuzzy_AHP and fuzzy_DS methods at 20 points with various solar radiation intensities and the number of dusty days parameters indicate that the fuzzy_DS method can more reliably determine the optimal PV farm locations. Additionally, as the fuzzy_DS method determines the optimal locations with different confident levels, this method can benefit decision-makers to determine the risks associated with selecting a specific site for constructing solar PV farms.

## Introduction

Given global warming and air-pollution induced by burning fossil fuels, utilizing renewable energies –such as solar energy- is indispensable in modern power networks. Solar energy as a clean, affordable, and sustainable source to generate electrical power is of great interest in arid and sim-arid regions. However, identifying the optimal location to benefit the maximum potential of solar energy is a big challenge.

There is a wide range of research papers that have attempted to extract the optimum location in multi-criteria decision-making (MCD) problems. For instance, voltage deviation and power loss^1–3^ have been chosen as a cost function and optimum location for generators that are used renewable energies have been identified. In such problems, the main objective is to maintain the power system at a proper operating level. Hence, in such problems, the researchers are facing an optimization problem and finding the optimum cost function value and maintaining technical constraints at a proper level is indispensable. As it is obvious, the cost function in these problems is known and explicit, however, the main question is that what is the proper method when we are encountering with an MCD problem? In MCD problems, the main objective is to extract the proper weights for each input layer.

To reach this goal, the geographical information system (GIS) techniques can be used to determine the optimal location for solar PV farms spatially^4^. Considering geographical, topographical and soil data, Xu *et al*.^5^ have determined potential locations for constructing coal-fired power plant sites using GIS. However, they considered only one sample point for each polygon; while, each parameter can vary in all geographical directions. GIS-based methods have been effectively used in different aspects of energy domain including renewable energy^6–9^, planning infrastructure projects^10,11^, energy demand estimation^12,13^, energy consumption modeling^14,15^, site planning of renewable energy powerplants^16,17^ and visual impact assessment^18,19^.

Anwarzai *et al*.^20^ utilized a multi-criteria decision analysis in GIS (GIS-MCD) to identify wind and solar energy capacities. Sarmiento *et al*.^21^ used a decision support tool to determine the solar radiation amount in Salta province, Argentina. Alavipoor *et al*.^22^ considered climatic parameters such as the number of dusty days, relative humidity, and topography parameters (slope and elevation) as input data in GIS to prepare maps showing appropriate sites for solar PV farms^22^.

GIS-based techniques can also be utilized to investigate the spatial variability of solar energy^23–26^. Wang *et al*.^27^ utilized satellite images with different spatial resolutions to determine the optimal location for constructing a nuclear power plant. Their results revealed the capability of Landsat-8 in comparison with other satellite imagery data. Although Wang *et al*.^27^ utilized red infrared to obtain more accurate results, they only considered a single parameter (temperature) to monitor the thermal plume of the nuclear power plant. Whereas, spatial data such as topography and climatic parameters are necessary to obtain more accurate results. Asakereh *et al*.^28^ have used a combination of fuzzy and AHP methods to determine an appropriate location for solar PV farms. Mierzwiak and Calka^29^ used a multi-criteria analysis to determine a suitable location for solar PV farms. Also, Idris and Abd Latif^30^ have used GIS multi-criteria decision-making systems to determine suitable areas for establishing power plants in Pahang, Raub. They have deployed fuzzy_AHP for allocating weights to different layers to produce a suitability map without considering the confidence level in their computations. Moreover, TOPSIS, AHP, and fuzzy methods can also be utilized to determine the optimal location in MCD problems^31,32^. In addition, Karimi *et al*.^33^ have considered some parameters such as land slope, elevation, distance from roads, distance from water resources, distance to faults, distance to rural regions, and land use to obtain optimum solution spatially. However, climatic factors that have a great impact on obtaining an optimum site for solar farms have not been investigated.

One of the main limitations associated with fuzzy_AHP or MCD systems is that these methods are not able to consider the uncertainty of model inputs into account. Despite the merits claimed in the literature, the methods used in the previous studies are able to produce a single suitability map without considering the uncertainty or confidence interval of produced results. Various climatic and socio-economic parameters need to be taken into account to determine suitable sites for constructing solar PV farms. As data related to each parameter might be obtained with different accuracy and different confidence level, the uncertainty of produced maps locating suitable sites for constructing PV farms needs to be taken into account. In fact, generating the suitability maps with a known confidence level would benefit management practices and decision-makers to select a specific site for constructing PV farms commensurate with their social and economic restrictions. In this case, the Dempster-Shafer (DS) theory, which is a generalized form of the Bayesian theory, proposes a set of principles to combine data from various sources to determine the uncertainty of input data^34^.

This study proposes a new framework combining the DS theory with a fuzzy system to identify the optimum solar farm sites in the Fars province, Iran. In the first step, a fuzzy system is used to homogenize the data from different input parameters, and in the second stage, the fuzzy outputs are introduced to AHP and DS systems. Finally, the accuracy of the generated map using the fuzzy_AHP (without considering the confidence level) will be compared with the generated maps using the fuzzy_DS method with confidence levels of 95%, 99%, and 99.5%, and capabilities of these two methods will be evaluated.

The contributions are this paper are summarized as follows:

1. This study proposes a new framework combining the DS theory and fuzzy system that incorporates a probabilistic uncertainty model to determine the uncertainty of the decision system and is able to generate suitability maps with desired confidence levels. According to the best author knowledge, in the power engineering system, this is the first study that considers the uncertainty of different sources in generating maps with different confidence levels showing the suitability of different areas for the construction of solar PV farms.
2. All input data is acquired in a real scenario, Fars province, Iran.
3. In this paper, different risk levels are investigated such that the operator will be able to select the proper output according to the available budget, load profile, etc.
4. In order to overcome burden time and boost solving procedure, a 4-pixel × 4-pixel windows is utilized.
5. Finally, to validate the fuzzy_DS method capability, its results are compared with those obtained by the fuzzy_AHP method.

## Results

In this section, the proposed method and numerical results are provided in subsection A and B, respectively. To do this, in subsection A the formulation of fuzzy_AHP, fuzzy_ANP methods, and the needed information about the selected case study are discussed. In addition, in subsection B, the prepared final maps that is obtained by these two methods are prepared and the applicability of the fuzzy_ANP method is compared with fuzzy_AHP method.

## Material and Methods

In this section, the case study features and proposed methods are discussed in subsection A.1, A.2, and A.3, respectively. To do this, in subsection A.1, to facilitate the reader to follow the case study, all needed information is described. Also, the formulation of the proposed method to be utilized in such multi-criteria decision-making problems is explained in subsection A.2 and A.3.

### Study area

**Figure 1:**
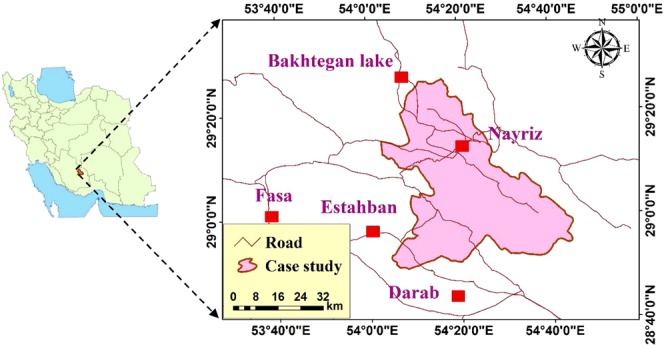
The geographical location of the study area in southern Iran.

This study focuses on southeastern Fars province, Iran, located between latitudes 28° 49′ and 29° 24′N and longitudes 54° 02′ and 54° 45′E, with an area of 124,444.671 km^2^ (Fig. 1). The highest and lowest altitude of the case study from the sea level is 1346 m and 2.830 m, respectively. The average temperature of the area during the study period (2010–2017) is 28.7 °C. The hottest days typically occur in July and the coldest days are in January resulting in an average temperature of 6.5 °C during this month. Most precipitation is in January with an average of 53 mm. It is relevant to note that average solar radiation in the selected case study is 3400 kWhm^−2^ year^−1^. Moreover, Fig. 1 depicts the case study geographically.

### Fuzzy_AHP method

The first step in the fuzzy technique is to determine the fuzzy sets. To do this, membership functions (MFs) are mathematically determined^35^. Moreover, Eq. (1) defines a fuzzy set^36^:

1 $$B=\{z,{\mu }_{B}(z)\}\,z\in Z$$

where *μ*_*B*_ is the MF, here a trapezoidal MF, to determine the degree of membership of *z* in fuzzy set *B*. Equation (2) shows a trapezoidal MF formulation. It is worth to mention that due to the nature of data, in this study a trapezoidal MF is found to be more appropriate.

2 $${\mu }_{B}(z)=g(z)=\{\begin{array}{ll}0 & z\le l\\ z-l/k-l & l < z < k\\ 1 & z\ge k\end{array}\}$$

In Eq. (2), *z* includes solar radiation intensity, air temperature, distance to major roads, land elevation, land use, relative humidity, and number of dusty days values and, at the same time, *l* and *k* are the boundary limits. According to Eq. (2), increasing the variable values lead to an increase in the MF value, indicating that these parameters can directly impact the solar radiation incident.

For cloudy days, distance to PTL, distance to residential areas and land slope, the following MF is used:

3 $${\mu }_{B}(z)=g(z)=\{\begin{array}{ll}1 & z\le k\\ k-x/k-l & l < z < k\\ 0 & z\ge k\end{array}\}$$

In Eq. (3), *z* is input data, and *l* and *k* determine the boundary limits for these parameters. According to Eq. (3), number of cloudy days, distance to PTL, distance to residential areas, and land slope have adverse effects on the efficiency of a solar farm.

As stated, the AHP method as a well-known MCD method is based on a pair-wise comparison matrix. In the AHP method, the problem is decomposed into a hierarchy of sub-problems. The AHP procedure can be summarized in six steps as below:

Step (1) In this step -the most important step- the problem is decomposed into a hierarchy of goals, criteria, sub-criteria, and alternatives.

Step (2) According to experts or decision-makers, data are classified into five classes including poor, low, moderate, good, and high. Then, the qualified classes are converted into quantitative numbers.

Step (3) A square matrix is formed in which the elements are defined as Eq. (4).

4 $$\{\begin{array}{c}{a}_{ii}=1\,diagonal\,elements\\ {a}_{ij} > 1\,If\,the\,criterion\,in\,the\,ith\,row\,is\,better\,than\,criterion\,in\,the\,jth\,column\\ {a}_{ij} < 1\,Otherwise\\ {a}_{ji}=\frac{1}{{a}_{ij}}\end{array}$$

Step (4) The weights are extracted from normalized eigenvector of the formed matrix.

Step (5) The consistency index (*CI*) is evaluated according to Eq. (5)

5 $$CI=\frac{{\gamma }_{\max }-m}{m-1}\,where\,{\gamma }_{\max }\,is\,the\,\max \,imum\,eignvalue$$

Step (6) The consistency ratio (*CR*) is calculated based on Eq. (6). It should be noted that *CR* should be less than 0.1^37^.

6 $$CR=\frac{CI}{RM}\,where\,RM\,is\,a\,random\,matrix$$

Prior to allocating weights to different layers and obtaining the final maps, the base maps in this study are initially fuzzified using fuzzy membership functions and then weighted accordingly. The weights for each factor are assigned with respect to pair-wise comparisons. The weights for each factor are assigned in accordance with their corresponding significance, which is determined based on an extensive literature review^32–40^. In the next step, the pair-wise comparison is made between the study factors to determine the final preference for each factor. The factors are then compared quantitatively and the pair-wise weight matrix for each factor is obtained.

### Dempster–Shafer (DS) method

The DS method, as a powerful MCD method, has the ability to analyze data while uncertainty is considered, enabling system planners to make proper decisions at different uncertainty levels^41^. The DS method is introduced by Dempster in 1976^34^. This method discusses the existing beliefs about a situation or a system. In this method, the belief intervals for the events can be different. Assume *θ* is a finite set of elements and is called the detection framework. An element can be system status, hypothesis or objective. Equation (7) describes *θ* and *Ω*(*θ(*(detection framework):

7 $$\theta =\{a,b,c\}\,{\rm{and}}\,\varOmega (\theta )=\{\phi ,\{a\},\{b\},\{c\},\{a,b\},\{a,c\},\{b,c\},\{a,b,c\}\}$$

The perfect status of the system is described by *Φ*.

*Φ* is an empty set that denotes the perfect status of the system. *A* = {*a*, *b*} is the subset of *θ* which means $A\subset {\theta }$. Therefore, *A* presents a system malfunction in *a* or *b*, and *θ* represents the system malfunction in *a*, *b*, or *c*^42^.

### Mass function, focal elements, and core elements

The mass function in the DS method is to show the reliability of the subset of decision-making frameworks^43^. These functions model all certainty existing for different situations. Equations (8–10) show the mass function definition:

8 $$m:\,\Omega (\theta )\to [0,\,1]$$

9 $$\Omega (\theta )=0$$

10 $$\sum _{A\subset \Omega (\theta )}m(A)=1$$

where *m* is the mass function and Eq. (8) means that the scope of this function is on the entire set of the detection framework that is (*Ω*^2^) and ranging between the closed interval of 0 and 1. The mass function calls a function of the basic probability assignment (*bpa*). The function *m*(*A*) represents the proportion of the share of the set *A* from all the relevant and available evidence and supports the claim about a certain element of *θ* that is owned by the set *A* (owned by set *A* not a peculiar subset of *A*).

In evaluating a condition in such a system suffers from a defect, *m*(*A*) can be represented as a degree of belief that can be obtained as the result of the observations related to a peculiar defect. There is the probability that the different evidence and information create various degrees of beliefs concerning the presented defect. Each subset of *A* from *θ* is called a focal element such that *m*(*A*) > 0. The core element, *c*, of the mass function in *θ* can be defined as^42^:

11 $$c={U}_{m(A)\ne 0}A$$

The belief function is defined as:

12 $$Bel(A)=\sum _{B\subset A}m(B)$$

$$Bel:\,\Omega (\theta )\to [0,1]$$

The plausibility function is defined as:

13 $$Pl(A)=1-Bel(\bar{A})=\sum _{B{\cap }^{}A\ne \Phi }m(B)$$

$$Pl:\,\Omega (\theta )\to [0,1]$$

*Bel*(*A*) function measures the probability that should be among the elements in *A*, indicating significant certainty of the belief *A* and the lower limit probability of *A*. The function *PI*(*A*) measures the maximum probability that can be distributed among the elements of *A*. the *PI*(*A*) describes the degree of the general belief related to *A* and is regarded as the upper limit function for probability *A*^42^.

### Belief interval

This space reflects the uncertainty belief space and the space size *PI*(*A*) - *Be*l(*A*) describes the unbeknownst related to *A*. Table 1 summarizes the meanings of different belief intervals.

**Table 1:** Various belief intervals with different meanings.

Interval	Description
[0, 0]	Certainly, no support can be made for the hypothesis
[1, 1]	The theory can definitely be supported
[0, 1]	The information is absolutely unknown
Interval	Supporting the theory and decision-making
[0.3, 1]	The theory could be supported
[0, 0.6]	The support from the theory is decreased

Both positive (positive relative probability) and negative (negative relative probability) weights are used in the proposed model, which are indicated as $\lambda (Tp)Eij$ and ${\rm{\lambda }}(\overline{{T}_{P}}){E}_{ij}$, respectively. The formulations for these two weights are shown in Eqs. (14) and (15), where *T*_*p*_ represents suitable pixels or cells for solar farms.

14 $${\rm{\lambda }}({T}_{P}){E}_{ij}=\frac{N(L{\cap }^{}{E}_{ij})/N(L)}{N({E}_{ij})-N(L{\cap }^{}{E}_{ij})/N(A)-N(L)}$$

, where *N(L∩E*_*ij*_) is the number of pixels labeled as suitable for solar farms in the class *E*_*ij*_. Moreover, N(L) shows the total number of pixels within the study that are suitable for solar farms. *N(E*_*ij*_*)* is the number of pixels in class *E*_*ij*_ and *N(A)* is the total number of pixels in the study area. The numerator and denominator in the Eq. (14) show suitable and unsuitable locations in class *E*_*ij*_, respectively. The negative target weight is defined as in Eq. (15).

15 $${\rm{\lambda }}(\overline{{T}_{P}}){E}_{ij}=\frac{N(L)-N(L{\cap }^{}{E}_{ij})/N(L)}{N(A)-N(L)-N({E}_{ij})+N(L{\cap }^{}{E}_{ij})/N(A)-N(L)}$$

The numerator indicates the ratio of suitable locations not within the *E*_*ij*_ class over the total number of locations in that class. The denominator shows the number of unsuitable regions outside class *E*_*ij*_. The negative weight in the control weight model is calculated by taking the natural logarithm of the relative probability in the Eq. (15). Overall, the relative probability ranges from 0 to infinity, thus, requiring both initial mass probability functions need to be normalized. The ratio of the probability for one feature over the sum of the probabilities for the entire features in all classes is then divided by the constant control variable *E*_*i*_, which not only facilitates normalization but also can be used as a measure of the relative significance of the features in each class.

16 $${\rm{m}}({T}_{P}){E}_{ij}=\frac{{\rm{\lambda }}({T}_{P}){E}_{ij}}{{\sum }^{}{\rm{\lambda }}({T}_{P}){E}_{ij}}$$

17 $${\rm{m}}(\overline{{T}_{P}}){E}_{ij}=\frac{{\rm{\lambda }}(\overline{{T}_{P}}){E}_{ij}}{{\sum }^{}{\rm{\lambda }}(\overline{{T}_{P}}){E}_{ij}}$$

18 $${\rm{m}}({\rm{\theta }})=1-{\rm{m}}({T}_{P}){E}_{ij}-{\rm{m}}(\overline{{T}_{P}}){E}_{ij}$$

**Figure 2:**
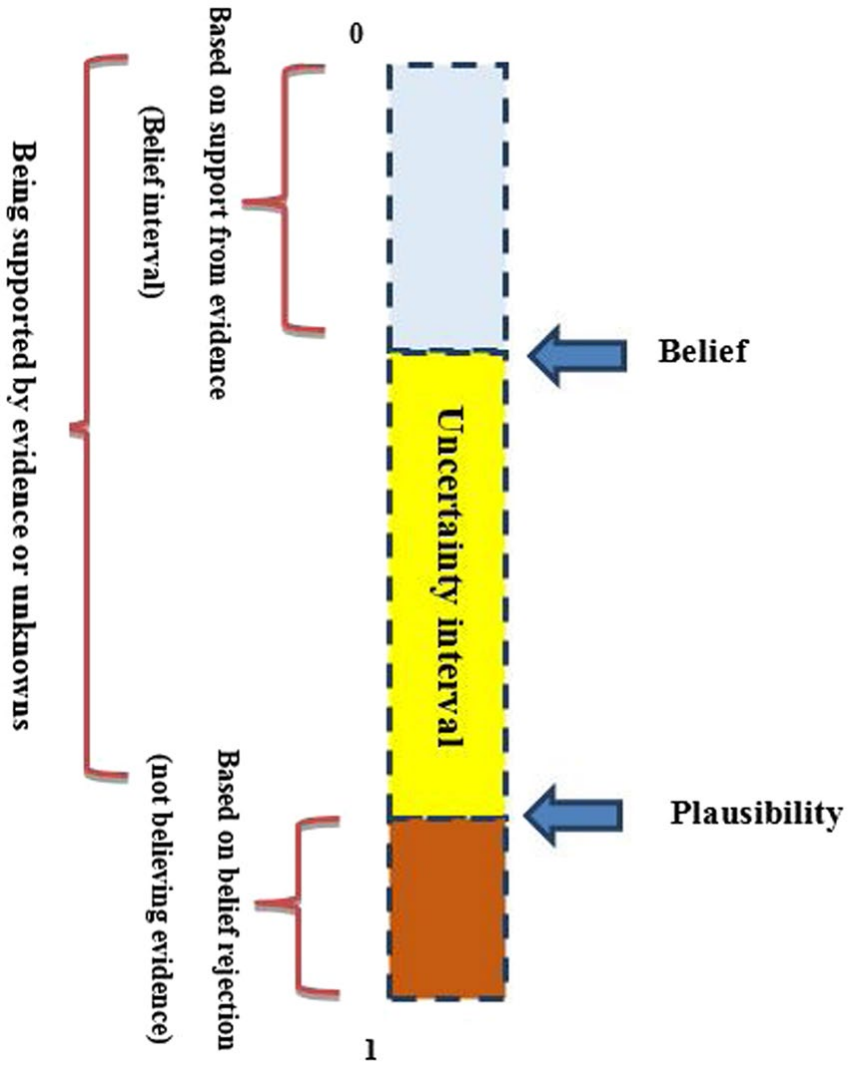
Uncertainty belief space.

According to Dempster Shafer Theory, the belief function *m(T*_*p*_*)E*_*ij*_, which is supporting the positive target proposition of *λ (T*_*p*_*)E*_*ij*_ can be derived directly from the mass functions. The disbelief function *m* $\overline{({T}_{p}}{E}_{ij})$ is supporting the opposing target proposition $\lambda (\overline{{T}_{p}}){E}_{ij}$ and can also be directly extracted from the mass functions, where $\overline{{T}_{p}}$ represents pixels or cells labeled as unsuitable for solar farms. The plausibility function m(θ) is obtained as the difference in magnitude between the belief and disbelief function and the constant 1 (Eq. (18)). Moreover, Eqs. (17) and (18) are employed to obtain the final suitability maps for solar farms. In addition, Fig. 2 represents the Uncertainty belief space in the DS method.

**Figure 3:**
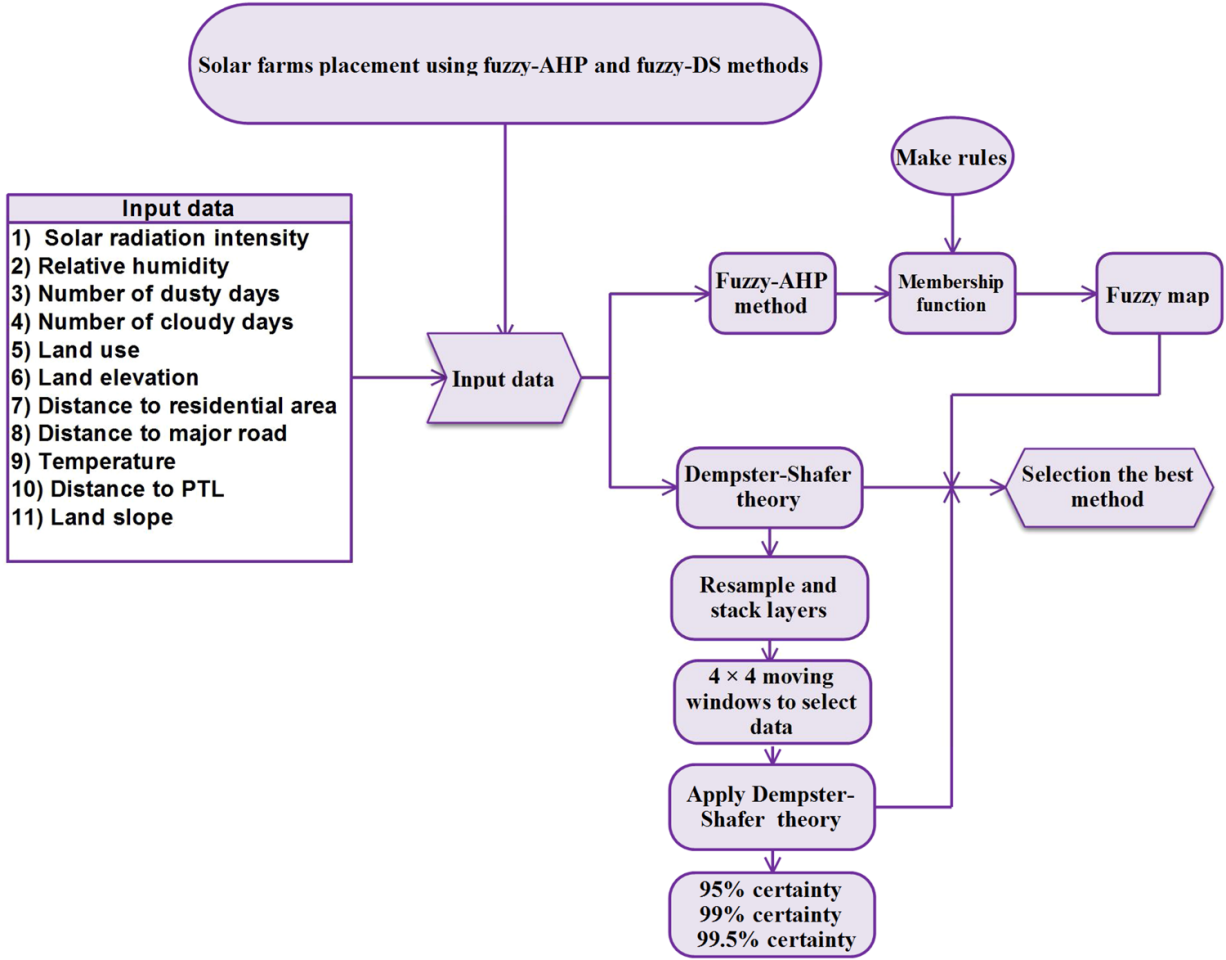
Flowchart for the methodology used in this region to determine the optimal solar farm site.

After preparing the weight map for each agent, the maps are aggregated and the final map is prepared. In this map, the weight of each pixel is calculated from the sum of the weights obtained for each factor. Lighter floors are not suitable for solar panel construction. Moreover, Fig. 3 shows the utilized steps in the fuzzy_AHP and fuzzy_DS methods for generating the suitability maps which subsequently are used for comparison of the two methods.

### An insight on computational methods

It is noteacible that the DS and AHP methods are different in terms of mathematical formulations, however as follows, the main drawback of them and how to overcome some disadvantages of AHP are explained.

As stated, AHP is a powerful method for solving complex problems including multi-criteria decision-making problems. In this method, the problem is decomposed into small constituent parts such that the proper weights get extracted through a series of pairwise comparision decisions. This method is used widely in many researches, however it suffers from a couple of minor issues such as mediocre accuracy and not considering uncertainty. The calculation speed, meanwhile in this is hindered due to this numerous pairwise comparison procedures. In addition, as stated in subsection A.2, the CR index must be smaller than 0.1 and if this index be greater than 0.1, the judgment must be repeated that can lower the speed more. The difference between these two methods, also, is summorized in as follows.

### Accuracy of the results

Pairwise comparison procedures are considered in AHP on which this method is merely applied in hierarchical mode, hence this method does not work as a network^44^ that means just outer dependencies are taken into account in this method. In many real occasions, however it is needed to study relations of inner dependencis among allternatives and parameters as well. The intreaction and feedback between criteria and alternatives are not indeed considered^45^. That is why this method is not accurate enough to be utilized in decision-making problems.

### Uncertainty

In real scenarios, uncertainty is unavoidable as stated above, however sometimes this factor is ignored because of the complexity that is added when it is applied in some decision-making methods such as AHP. It is worth mentioing that, in the DS mehtod as a generalization of the Bayesian theory needs to be interpreted such that its factor to show the uncertainty issue is denoted by “basic probability assignment” (bpa) parameter (see Eqs. (8–10)). In order to facilitate the comparison of the mentioned methods, the main features of AHP and DS are qualitatively described in Table 2.

**Table 2:** The summorized features of AHP and DST methods.

Method	Accuracy	Uncertainty
AHP	Avarage	Not considered
DST	high	Considered

## Numerical Results

In order to better describe the advantages of the fuzzy_DS method, how to prepare input layers is initially presented in subsection B.1. In addition, fuzzy_AHP implementation and fuzzy_DS method procedures are described in subsection B.2 and B.3, respectively. It is worth mentioning that all simulations are run using ArcGIS V10.2 on a Laptop (2.6 GHz, 6 GB RAM).

### Preparing layers

As stated, eleven parameters including solar radiation intensity, air temperature, distance to power transmission line (PTL), distance to major roads, land slope, distance to residential areas, land elevation, number of cloudy days, relative humidity (RH), land use, and number of dusty days are considered as input parameters^46^. The following steps are performed to extract the input parameters:

- The geological maps in 1:100,000 scale is used to derive lithology and fault maps.
- The topographical map on the scale of 1:25,000 is used to provide roads and rivers maps.
- The digital elevation model (DEM) is extracted from the shuttle radar topography mission (SRTM); thereafter; the slopes are determined based on DEM.
- Climatic parameters including number of dusty days, number of cloudy days, relative humidity, air temperature, and solar radiation intensity from 2010 to 2017 are used^46^ for computations.
- Land use map is obtained from Landsat 7 ETM + satellite images.

**Figure 4:**
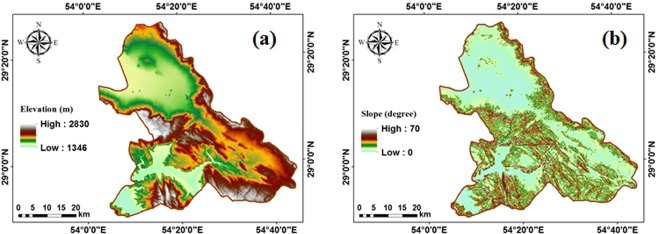
Topographic parameters (**a**) DEM map and (**b**) land slope map in the study area.

Figure 4 shows DEM and land slope maps. As shown in Fig. 4a, the land elevation in most parts of the study area is lower than 1500 m. In addition, the minimum and maximum slopes are between 0 and 70 degrees, respectively with the greatest slopes in central and southern (Fig. 4b).

Table 3 classifies^22^ the suitability of land elevation and slope sites being used as a solar farm. As Table 3 shows, sites with land elevations and slopes respectively greater than 750 m and 4 degrees are not economically suitable to be used as solar forms^22^.

**Table 3:** Suitability of land elevation and slope for sites being used as solar farms^22^.

Parameters	Class	Description
Elevation (m)	>750	Suitable
	<750	Not suitable
Slope (degree)	<1	Suitable
	1–4	Fair
	> 4	Not suitable

**Figure 5:**
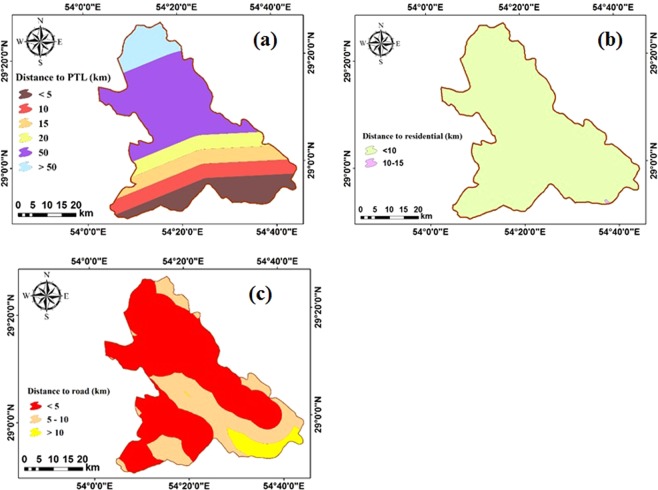
(**a**) distance to PTL, (**b**) distance to residential, and (**c**) distance to road maps in the study site.

In order to prepare maps associated with the distance from major roads, residential areas, and PTL, the buffer tools in ArcGIS are used (Fig. 5). The PTL map is categorized into six regions (Fig. 5a). As seen, southern areas are located in less than 5 km from PTS, while, this distance is more than 50 km in the northern parts. In addition, distance to residential in almost regions of the study site is lower than 10 km that would be suitable to construct solar PV farms (Fig. 5b). Distances to major roads that are between 5 and 20 km are classified into three classes of less than 5 km, between 5 and 10 km, and greater than 10 km (Fig. 5c).

**Figure 6:**
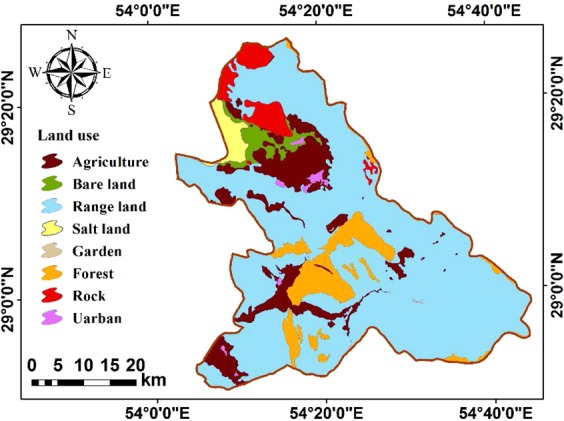
Land use map.

The land-use map of the study site is categorized into nine classes including urban, forest land, rangeland, woodland, bare land, agricultural areas, salty land, garden, and sand dune (Fig. 6). It is worthwhile mentioning that bare lands are the most suitable areas to utilize solar energy^32^.

**Figure 7:**
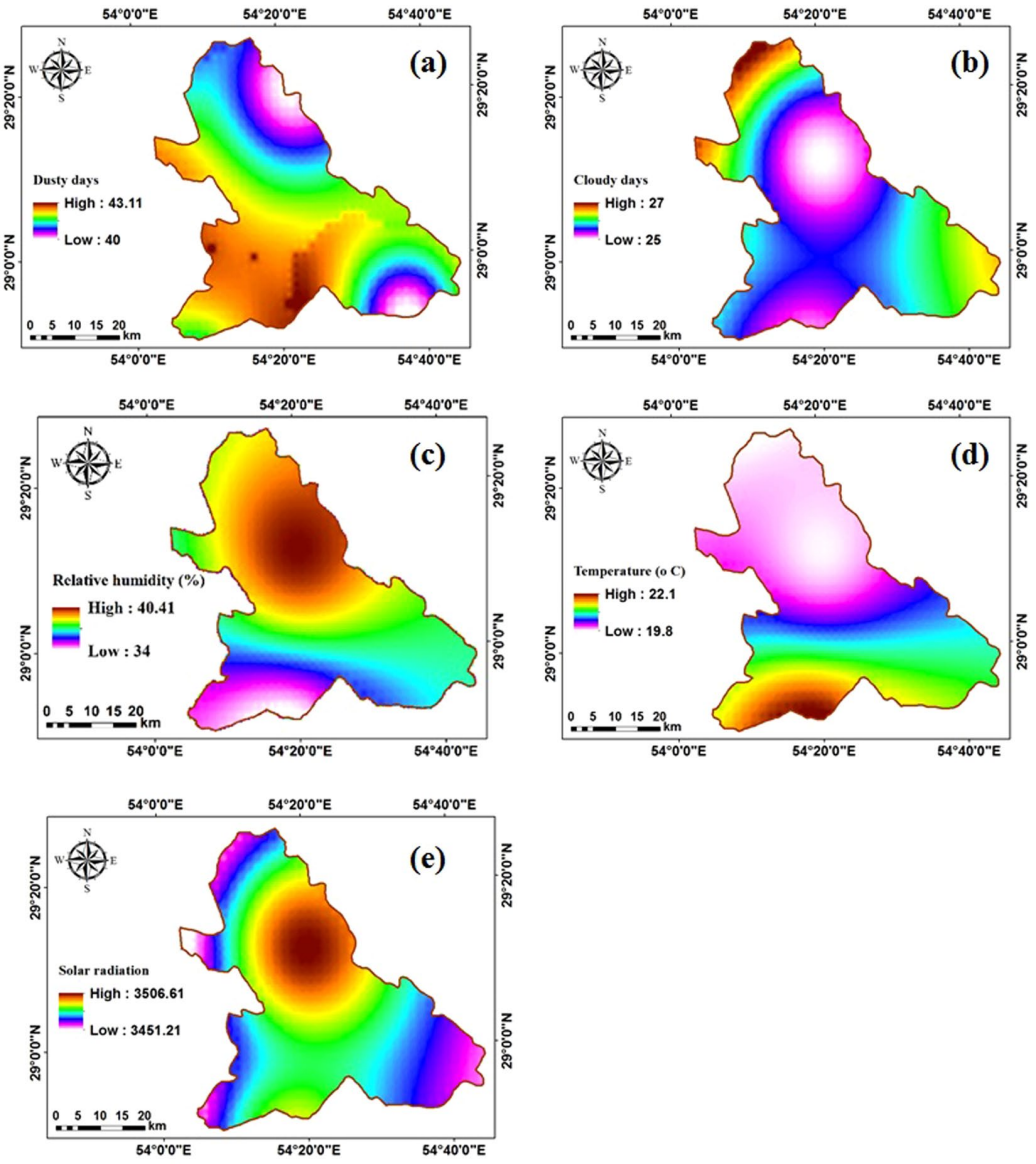
Climatic maps. (**a**) dusty days, (**b**) cloudy days, (**c**) relative humidity, (**d**) temperature, (**e**) solar radiation.

In addition to topography maps, climatic parameters are also used to determine the optimal location of PV farms more accurately. Among climatic parameters, five parameters including number of dusty days, number of cloudy days, relative humidity, air temperature, and solar radiation intensity are more influential^32^. To determine the interpolation maps, the ordinary kriging (OK) method in ArcGIS software is used (Fig. 7). According to Fig. 7a, number of dusty days, as an unsuitable parameter in receiving solar energy, has a higher value in southern parts, whereas it is lower in the northern areas of the study site. Number of cloudy days in northern parts is more than the southern and central parts (Fig. 7b). According to Fig. 7c, relative humidity in the south of the study site is the highest, while, the temperature in the south is moderate (Fig. 7d). Moreover, solar radiation intensity as an important parameter for solar PV farms is high in the northern areas (Fig. 7e).

### Fuzzy_AHP method results

In order to apply the fuzzy method, firstly, input parameters are homogenized (see Fig. 7). It is worth mentioning that the needed data to form fuzzy maps are formed according to Table 4. Also, Eqs. (2) and (3) are utilized to prepare MFs. As the membership function value is increased from 0 to 1, the suitability for constructing solar PV farms is also increased and vice versa.

**Table 4:** Fuzzy limits considered for each parameter.

Parameters	Suitable range
Solar radiation intensity (kWhm^−2^ year^−1^)	>1900
Distance to power transmission line (km)	<5
Distance to major roads (km)	<5
Distance to residential areas (km)	<10
Land elevation (m)	>1200
Land slope (degree)	<1
Land use	Bare land
Number of cloudy days	<30
Relative humidity (%)	<26
Number of dusty days	<40
Air temperature (°C)	>24

A fuzzy membership function (MF) value of 1 assigned to the parameters having the limits determined in Table 4, and fuzzy membership values for the values greater or less than Table 4 limits are determined based on Eqs. (2 and 3).

**Figure 8:**
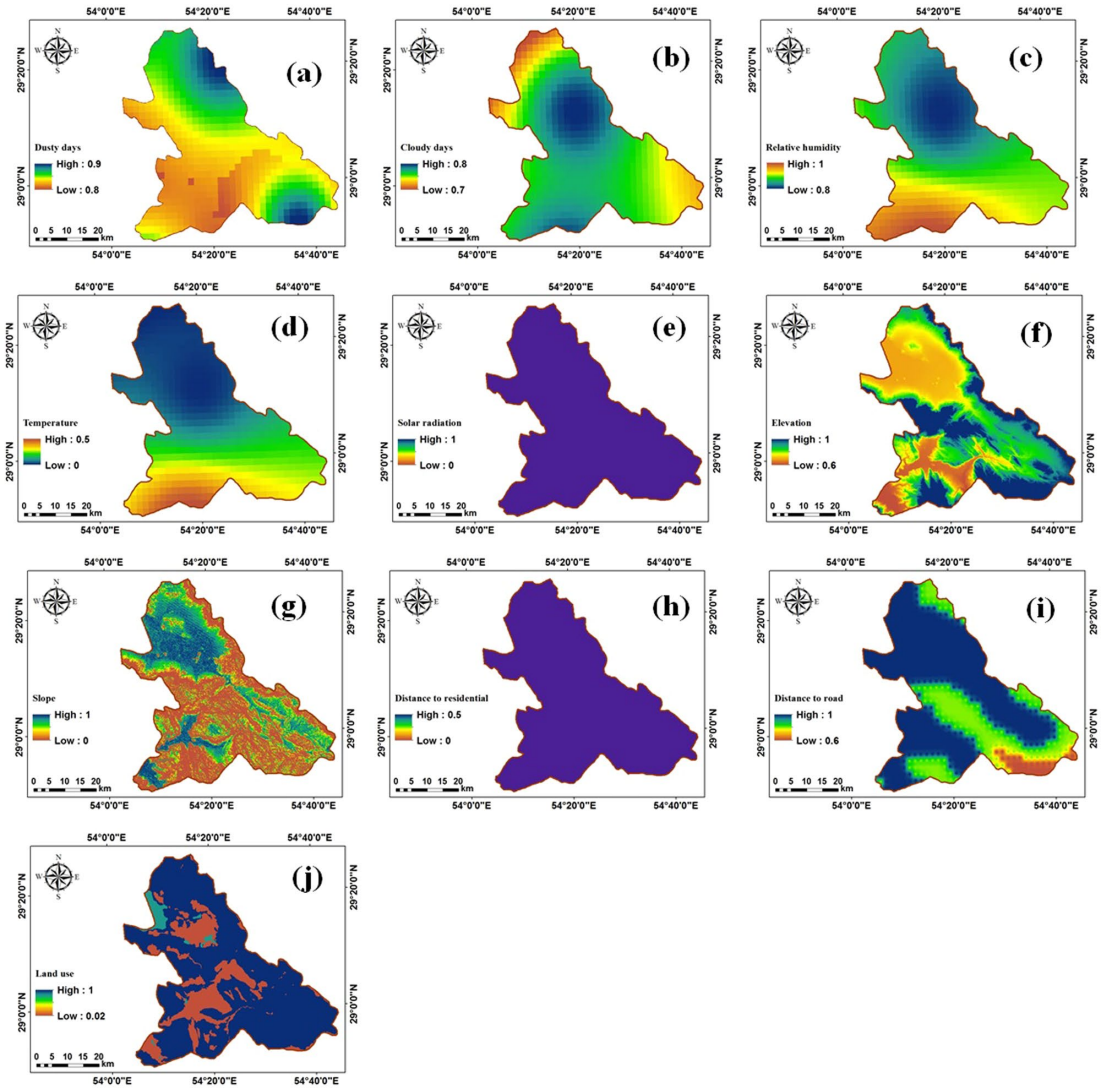
Fuzzy maps for different input parameters including (**a**) dusty days, (**b**) cloudy days, (**c**) relative humidity, (**d**) air temperature, (**e**) solar radiation intensity, (**f**) DEM (**g**) land slope degree, (**h**) distance to PTL, (**i**) distance to residential areas, (**j**) distance to major roads, and (**k**) land use.

According to Fig. 8a, the MF values for the parameter associated with number of dusty days are between 0.8 and 0.9 indicating the high suitability area for being used as solar farms. The northern and southeastern that have erosion-sensitive formations experienced more dusty days.

The number of cloudy days as an unsuitable parameter that blocks solar energy is lower in the southern and northeastern parts (Fig. 8b). As shown in Figs. 8c and 4d, higher relative humidities and air temperatures occurred in northern parts. Due to the proximity to Bakhtegan Lake, the temperature and humidity in the southern area are low and high, respectively (Fig. 8c,d).

Solar radiation intensity and distance to residential areas are high in all parts of the study site (Fig. 8e,i). In fact, the solar radiation intensity in the entire study area is more than 3400 kWhm^−2^ year^−1^. Moreover, The elevation and slope in the study area are shown in Fig. 8f,g, respectively. Based on Fig. 8h, the MFs assigned to distance to power transmission line (PTL) parameter has greater values in southern areas, indicating that it would be easier to transfer the generated electrical power in southern parts than other areas. Figure 8(i–k) show the fuzzy maps associated with distance to residential areas, major roads, and land use parameters, respectively.

**Figure 9:**
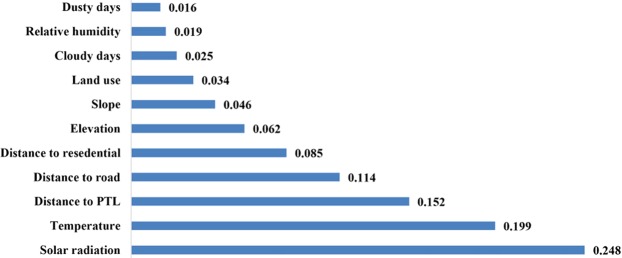
The weights associated with each parameter obtained by the AHP method.

After preparing the fuzzy maps, it is important to determine the most affecting parameter that can influence the suitability of a site for being used as a solar PV farm. Figure 9 depicts the weights associated with each parameter according to the AHP method.

**Figure 10:**
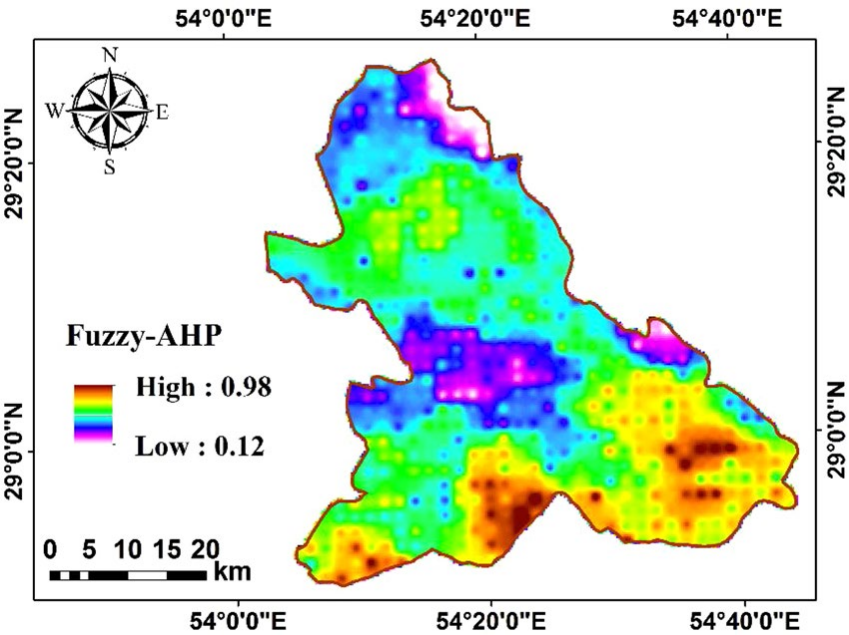
Fuzzy_AHP map.

According to Fig. 9, number of dusty days and solar radiation intensity have the smallest and the greatest effects on the suitability index, respectively. Thereafter, the obtained weights are used to generate an overall fuzzy_AHP map (Fig. 10).

The fuzzy_AHP method yields normalized suability indices ranging between 0 (lowest suitability) and 1 (highest suitability) indicating the optional performer of the area for being used as solar PV farms. Different regions of the study area are classified into five suitability classes of poor with suitability indices ranging between [0, 0.2), low [0.2, 0.4), moderate [0.4, 0.6), good [0.6, 0.8), and high [0.8, 1]. The fuzzy_AHP method obtains indices between 0.12 and 0.98 (Fig. 10). As seen in Fig. 10, most parts of the study area are categorized as moderate to good (blue and green areas in Fig. 10) for construction solar PV farms. The fuzzy_AHP method indicates high suitability indices of around 0.98 (areas with brown color in Fig. 10) in southern and southeastern parts of the study site which experiences suitable solar radiation intensity, high air temperatures, and low relative humidity.

### Fuzzy_DS method results

The main purpose of this section is to analyze and differentiate suitable areas for solar farms construction using the DS method at different confidence levels. For this aim, input parameters are selected as *E*_*i*_ *(i* = *1, 2, … L)*. The results of combining the input layers and calculating the weight of each floor are as Table 5.

**Table 5:** Characteristics of each layer class in the Dumpster Schafer method.

layer	category	number of pixels	pixel of solar farms	*m(Tp)*	*M* $(\overline{Tp})$	*m(θ)*
Distance to a power transmission line (km)	<5	47894	3105	0.006	0.179	0.815
	5–10	43797	37296	0.577	0.074	0.349
	10–15	37476	29167	0.417	0.092	0.491
	15–20	31485	851	0.004	0.172	0.824
	20–50	122897	0	0.000	0.310	0.690
	>50	28885	0	0.000	0.173	0.827
Distance to residential areas (km)	<10	312235	70416	1.000	0.050	−0.050
	10–15	199	0	0.000	0.950	0.050
Distance to major roads (km)	<5	208769	40268	0.090	0.436	0.475
	5–10	87034	28644	0.441	0.242	0.317
	>10	16631	1507	0.469	0.323	0.208
land use	Agriculture	37677.92	9021.109	0.002	0.127	0.871
	bare land	7249.68	0	0.000	0.132	0.868
	range land	220315.4	54883.14	0.000	0.089	0.910
	salt land	6228.599	0	0.000	0.132	0.868
	garden	229.7434	120.1631	0.986	0.128	−0.114
	forest	26820.41	6266.233	0.002	0.128	0.870
	rock	12142.36	0	0.000	0.135	0.865
	urban	1769.875	128.3561	0.009	0.129	0.862
Number of dusty days	>40	312434	70419	1.000	0.000	0.000
	<40	0	0	0.000	1.000	0.000
Number of cloudy days	>30	0	0	0.000	1.000	0.000
	<30	312434	70419	1.000	0.000	0.000
Relative humidity (%)	<30	0	0	0.000	0.416	0.584
	30–40	312253	70400	0.001	0.168	0.831
	>40	181	19	0.999	0.416	−0.415
Air temperature (°C)	>24	0	0	0.000	1.000	0.000
	<24	312434	70419	1.000	0.000	0.000
solar radiation	>1900	312434	70419	1.000	0.000	0.000
	<1900	0	0	0.000	1.000	0.000
Land slope (degree)	<1	24551	4426	0.499	0.331	0.169
	1–20	238544	52286	0.066	0.362	0.572
	>20	49339	13712	0.435	0.306	0.259
Land elevation (m)	<1200	0	0	0.000	0.264	0.736
	1200–2000	204959	55453	0.324	0.147	0.529
	2000–2500	94608	14675	0.348	0.312	0.340
	>2500	12873	296	0.328	0.277	0.395

According to Table 5, it is found that the cells in the proper locations to be the host of solar farms have more m (θ) values than the other pixels.

**Figure 11:**
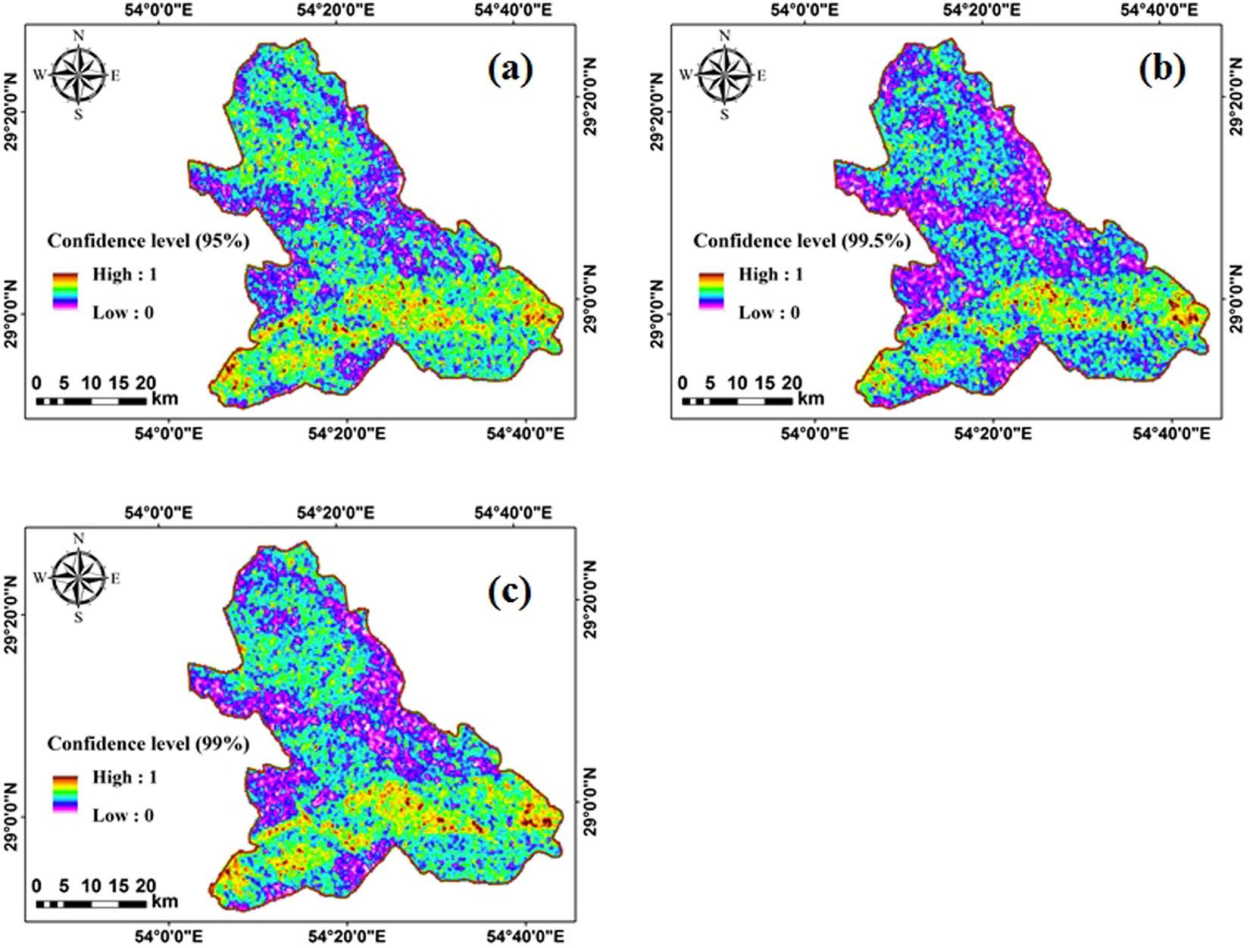
The fuzzy_DS method results with different confidence levels of (**a**) 95%, (**b**) 99%, and (**c**) 99.5%.

As stated, the main challenge in the DS method is burden time. Hence, to boost the DS method solving procedure, in this paper, 4-pixel × 4-pixel windows are selected on the image. Thereafter, for each 16 pixel and 12 bands, the DS method is implemented and the results are saved as new images. This process is continued until the 4-pixel × 4-pixel windows covered the entire image (entire study area). Moreover, Fig. 11 depicts the results obtained by the fuzzy_DS method.

As it is obvious from Fig. 11, by increasing the confidence level, the area of regions with a high suitability index decreased. As seen, the southern parts are found to be suitable in all confidence levels.

The suitability of different regions in the study site is classified into five classes including poor, low, moderate, good, and high. Table 6 summarizes the percentage of areas categorized by fuzzy_AHP and fuzzy_DS methods. As Table 6 shows, the fuzzy_AHP model rank about 36% of the study area as unsuitable regions, whereas the fuzzy_DS method categorized the majority of area (60%) as low and moderately suitable in all confidence levels. It is worthy to note that by increasing the confidence level, the fraction unsuitable areas increases.

**Table 6:** Percentage of areas covered by different suitability classes determined based on fuzzy_AHP and fuzzy_DS methods.

Class	Fuzzy_DS method			Fuzzy_AHP method
	95%	99%	99.5%	
Poor (0–0.2)	19.97	26.20	28.58	10.62
Low (0.2–0.4)	34.63	32.30	31.50	25.03
Moderate (0.4–0.6)	26.84	24.80	23.60	31.83
Good (0.6–0.8)	14.15	12.90	12.60	20.90
High (0.8–1)	4.41	3.80	3.72	11.62

Generating maps with different confidence levels would enable the management practices to determine the suitable zones for establishing the solar PV farms in compliance with their socioeconomic targets of the region. A confidence level of 95% can be used for regions with a prosperous economic perspective. However, when investments are limited and the objective is to ensure high performance, a map with a high confidence level (99.5%) is recommended to restrict the risk of a low return on investments.

Although the fuzzy_AHP method can be used to perform pair-wise comparisons and assign weights to each layer and ultimately generate the final map, the fuzzy_DS model is able to generate the final map based on the target level of confidence –as a straightforward feature- which cannot be obtained by the fuzzy_AHP system.

## Further Discussion

As stated before, one of the most important features of DS is considering uncertainty in decision-making problems. By applying the uncertainty, known also as confidence level, the system planner indeed will be able to make a proper decision according to the existing conditions, budget, etc. The main question, however is that how accurate DS method can extract the results in different confidence levels. To illustrate this issue in this subsection, we provide an extra comparative disscusion. It is noteacibe that in order to faciltate comparing process, four classes are considered for each input parameters as shown in Table 7 ^32^.

**Table 7:** Standard values for different input parameters in the literature.

Parameters	Very High suitable range	High suitable range	Moderate suitable range	Low suitable range
Solar radiation intensity (kWhm^−2^ year^−1^)	>2100	2100–2000	2000–2100	<1900
Distance to power transmission line (km)	<5	5–10	10–15	>15
Distance to major roads (km)	<5	5–10	10–15	>15
Distance to residential areas (km)	<10	10–12	12–15	>15
Land elevation (m)	>1200	1000–1200	750–1000	<750
Land slope (degree)	<1	1–2	2–3	>3
Land use	Bare land	Farming land	Agriculture land	Wet land
Number of cloudy days	<30	30–40	40–50	>50
Relative humidity (%)	<26	26–36	36–50	>50
Number of dusty days	<40	40–45	45–50	>50
Air temperature (°C)	> 24	22–24	22–24	<20

Each parameter has the highest suitablity in 4^th^ class. As it is obvious from Table 8, the best location is the place that the total value of classes is 44. In the selected case study, however the highest total class value is 39. For comparison, twenty locations through the entire case study are chosen such that the class value and its summation total value are calculated and prepared in Table 8.

**Table 8:** Parameters’ classification results according to the chosen twenty points.

DS method			Parameters											Net classes
95%	99%	99.5%	#1	#2	#3	#4	#5	#6	#7	#8	#9	#10	#11	
0.41	0.34	0.27	4	1	2	1	1	4	1	1	1	4	1	21
0.85	0.79	0.74	4	4	3	4	4	4	4	4	3	4	1	39
0.8	0.7	0.68	4	4	3	4	3	4	4	3	3	4	1	37
0.48	0.41	0.32	4	1	3	1	1	4	2	1	1	4	1	23
0.6	0.48	0.44	4	2	3	1	1	4	1	2	2	4	1	25
0.45	0.35	0.29	4	1	2	1	2	4	1	1	1	4	1	22
0.47	0.38	0.3	4	1	2	1	2	4	1	1	1	4	1	22
0.53	0.43	0.37	4	2	3	1	2	4	1	1	1	4	1	24
0.63	0.5	0.48	4	2	3	1	3	4	2	1	1	4	1	26
0.65	0.53	0.5	4	2	3	1	3	4	2	1	2	4	1	27
0.68	0.55	0.51	4	2	3	1	3	4	4	1	1	4	1	28
0.72	0.6	0.55	4	2	3	4	3	4	4	1	1	4	1	31
0.73	0.63	0.57	4	2	3	4	3	4	4	2	1	4	1	32
0.74	0.64	0.59	4	2	3	4	3	4	4	3	1	4	1	33
0.76	0.65	0.6	4	2	3	4	3	4	4	3	2	4	1	34
0.77	0.67	0.62	4	4	3	4	3	4	4	1	3	4	1	35
0.79	0.69	0.63	4	4	3	4	3	4	4	3	2	4	1	36
0.69	0.57	0.52	4	2	3	3	4	4	2	1	1	4	1	29
0.7	0.59	0.53	4	2	3	4	4	4	2	1	1	4	1	30
0.83	0.76	0.7	4	4	3	4	4	4	4	3	3	4	1	38

According to Table 8, the total class value is proportional to the suitablity (and vice versa). Hence, when one person approaches the south, the suitability in all confidence levels is increased, however, there is a slight difference between confidence levels. In addition, for in all points, the suiatility index for confidence level of 95% is the highest because of considering more uncertainty value.

**Figure 12:**
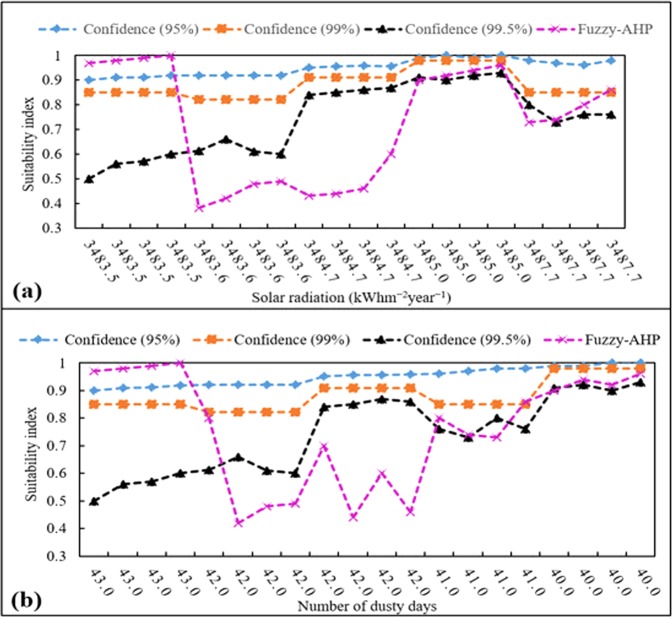
Suitability indices determined by the fuzzy_AHP and fuzzy_DS methods at 20 randomly selected points with different (**a**) solar radiation intensities (**b**) number of dusty days.

In order to compare the fuzzy_DS and fuzzy_AHP methods, suitability indices for areas with the different number of dusty days and different solar radiation intensities (having the lowest and the highest AHP weights, respectively) are compared. Hence, the yielded suitability index by the fuzzy_DS and fuzzy_AHP are compared in 20 randomly selected points. Figure 12a depicts the location suitability index at 20 randomly selected points and their associated solar radiation intensity.

It is expected that regions with higher solar radiation intensities yield higher suitability indices. Although fuzzy_DS graphs with confidence levels of 95% and 99% show a rather increasing trend in suitability index by increasing the solar radiation intensity, the fuzzy_AHP method depicts random fluctuations between 0.38 and 0.98 without a specific trend (Fig. 12a). The fuzzy_DS graph with a confidence level of 99.5% also shows an overall increasing trend with increasing solar radiation intensity. However, the more stringent criteria due to the use of a high confidence level yield relatively lower suitability indices compared to 95% and 99% confidence levels.

Similarly, it is expected that solar farms located in dusty areas receive less solar radiation intensities, thus have lower suitability indices compared to areas with less dust. As seen in Fig. 12b, the fuzzy_DS graphs show an overall increasing trend of suitability index by decreasing number of dusty days. However, the fuzzy_AHP method represents large random fluctuations without a specific trend (Fig. 12b). This is due to the fact that the fuzzy_AHP method cannot effectively handle the spatial variability of uncertainty associated with measurements of input parameters across the study site. Although the fuzzy_AHP method has been used to determine suitable locations for solar PV farms in several studies^28,29,47–51^, the fuzzy_DS method that considers uncertainty can more reliably be used for this purpose.

The main drawback associated with the fuzzy_DS method in comparison with the fuzzy_AHP method is its significantly longer computation time. The average computation time for the fuzzy_DS method (even by using 4-pixel × 4-pixel windows) is about 5 times longer than the fuzzy_AHP method. Hence, this study recommends a 4-pixel-by-4-pixel window for computation at each time step to increase the computation speed compared to computation with a 1-pixel-by1-pixel window. It is worth mentioning, generally, calculation and complexity in this problem are not important, because we can do all the processes offline. So, we have enough time to complete the decision-making process. This is related to our technical issue which can be an offline planning process, instead of the operation. Nevertheless, the DS model in conjunction with fuzzy membership functions increases the robustness of computed results.

## Conclusions

Given the advantages of solar energy in comparison with fossil fuels to generate electrical power, this study proposed a method to determine the optimal location for constructing PV farms. To do this, eleven parameters including solar radiation intensity, air temperature, distance to PTL, distance to major roads, distance to residential areas, land elevation, land slope, land use, number of cloudy days, relative humidity, and number of dusty days are considered as input parameters. The fuzzy method with trapezoidal membership functions is used to homogenize the input parameters. Thereafter, AHP and DS methods were utilized to prepare final maps depicting the optimal PV farm locations to maximize the utilization of solar energy.

Finally, southeast of Fars province located in Iran as a sim arid area was selected and both fuzzy_AHP and fuzzy_DS methods were independently applied to find the optimal location. Results showed the south of the study site located in southeastern Fars province, Iran could be an appropriate place for being used as a solar PV farm. It is worth mentioning that in the fuzzy_DS method, by increasing confidence level in the fuzzy_DS method the area of suitable location in the study sites for a solar PV farm decreased.
